# The biomechanical significance of the elongated rodent incisor root in the mandible during incision

**DOI:** 10.1038/s41598-022-07779-z

**Published:** 2022-03-09

**Authors:** Philip J. R. Morris, Philip G. Cox, Samuel N. F. Cobb

**Affiliations:** 1 Hull York Medical School, University of Hull, Hull, HU6 7RX UK; 2 Hull York Medical School and Department of Archaeology, University of York, York, YO10 5DD UK

**Keywords:** Biomechanics, Evolution

## Abstract

Rodents are characterised by a distinctive masticatory apparatus which includes a single pair of enlarged and continually growing incisors. This morphology, termed diprotodonty, has also independently evolved in a number of other mammals, including the aye-aye. This study examined the functional significance of the internal “root” of the elongated rodent-like incisor. The mandibles of four rodents and an aye-aye were modelled to exhibit incrementally shorter incisor roots. Finite element analysis was used to predict stress and strain patterns across the jaw to determine whether the length of the incisor root contributes to the resistance of mechanical forces encountered in the mandible during incision. It was found that von Mises stresses increase in the region of the mandible local to where the incisor is removed, but that the stress distribution across the wider mandible is only minimally affected. Thus, the long internal incisor appears to play a small role in resisting bending forces close to the incisor alveolus, and may act with the arch-like mandibular shape to strengthen the mandible in this region. However, the impact across the whole mandible is relatively limited, suggesting the highly elongate incisor in diprotodont mammals may be principally driven by other factors such as rapid incisor wear.

## Introduction

Rodentia is the most speciose order of mammals, containing over 2500 extant species^1^, all possessing a highly specialised and distinctive masticatory apparatus including a pair of elongated, curved and continually growing incisors. This condition, known as diprotodonty, is not, however, unique to rodents, having evolved independently in a number of other mammalian groups including lagomorphs, hyraxes, marsupials, and primates^2,3^. The aye-aye (Primates) in particular has a masticatory apparatus very similar to that of rodents, with a pair of continually growing incisors that lack enamel on their lingual side and form a sharp chisel-like groove with wear^4^. These are used to gnaw through bark to obtain xylophagous insect larvae^5,6^. The skull and mandible of the aye-aye have been shown to be morphologically convergent with rodents, specifically squirrels^7^. This may indicate strong functional drivers of morphology in these taxa, in particular, the need to produce high bite forces at the incisors, which themselves exhibit features related to the resistance of forces encountered during gnawing^8–10^. Whether the presence of a long rodent-like incisor within the mandibular body, in conjunction with bony adaptations of the mandible, plays a role in the mechanical adaptation of the mandible to resisting bending during incisal biting is of key interest in understanding the success of rodents, and the independent evolution of diprotodonty in other non-rodent taxa.

During gnawing, the incisors of diprotodonts are exposed to bending and shearing stresses^11^, and, in order to function, incisors must be able to resist the forces encountered. Such a need to resist bending means that diprotodont incisor morphology varies in a way that is largely predictable from ecology and mechanics^9,12–14^. During a bite, forces encountered at the tooth tip lead to stresses and strains in the root, the periodontal ligament (PDL), and the surrounding bone^15–17^. Rodent incisors are very long and deep rooted, and possess only labial enamel, with just their embedded lingual side covered by PDL^18^. It has been proposed that a longer incisor may play a role in the dissipation of stresses within the rodent mandible^19^. However, it would not be expected that forces propagate along the entire incisor length, as rodent incisors are continually growing (hypselodont) teeth, and therefore technically rootless, with the apical portion of the incisor consisting principally of newly formed unmineralised tissues^18^. For simplicity the portion of the tooth which projects into the bone is referred to as the root in this paper. The question of whether a deep tooth root plays a mechanical role in the resistance of forces encountered during biting has been tested in *Thylacosmilus atrox*, with their long-rooted maxillary canines, and found to have a very minor impact on the cranial biomechanics in a series of simulated bite scenarios^20^. The impact of an elongated tooth root on the resistance of forces however has not been tested in the mandible, which is both more compliant than the cranium and, in the case of rodents, can occupy most of the mandibular corpus.

In rodents, the unerupted portion of the incisor typically extends distally to the end of the tooth row or just beyond^9^, occasionally extending as far as, or into, the condylar process^21^. It has previously been suggested that the deep internal projection of the incisor within the rodent mandibular body is responsible for the arch-like shape of the mandible visible in many rodents and a reduction in the bending moment by up to 25% compared to a beam-like mandible^3,22^. In addition, other biological curved structures such as long bones have been proposed to make the location and orientation of strains more predictable^23,24^.

The principal focus of this study is whether, in the mandibles of diprotodont mammals, the replacement of bone with the relatively stiffer dental tissues of the incisor acts to increase the mandible’s ability to resist bending. A series of four rodents representing the major morphological and phylogenetic groups, and one non-rodent diprotodont, the aye-aye, will be modelled with incrementally shorter incisor roots and examined using finite element analysis (FEA^25^) to assess the resulting differences in stress and strain distributions. The core aim of this study is to determine whether the length of the incisor root is functionally significant for the resistance of mechanical forces encountered during incision. Three main hypotheses will be tested:

1. *Reduction in the length of the incisor root will result in an increase in stress across the mandible.* Specifically, we expect to see this most clearly in von Mises stresses along the ventral surface of the mandible based on the assumptions that the rodent-like mandible operates in a manner similar to an arch^22^, and that an arch operates in compression. We will test this by comparing the stress magnitudes predicted by the FE models with complete incisors and those with virtually shortened roots.
2. *The incisor grows through an area of naturally low strain.* This will be assessed by creating FE models in which the incisor length is reduced and the resulting space filled with bone. In particular, we wish to explore whether the area from which the incisor has been removed will experience lower strains than the area immediately around it, and whether the path that would have been filled by the incisor is visually apparent in the strain contour maps. We predict that the path through which the incisor grows will directly coincide with a region of reduced strains, and therefore bone resorption, to accommodate the growth of an elongated structure.
3. *The non-rodent diprotodont aye-aye will perform in a mechanically similar way to the rodents, but will produce more extreme results*. We anticipate the aye-aye will exhibit patterns of stress similar to those observed in other examined diprotodonts, in particular the grey squirrel, but that it will experience stress magnitudes that are far higher than in any of the rodent taxa. We predict similarity with the grey squirrel based on previous results which indicate the aye-aye has convergently evolved a masticatory apparatus resembling sciurid rodents^7^, and expect a finding of more extreme stress values based on previous results that indicate the aye-aye mandible follows the same pattern of variation in incisor form as rodents but does so in a more exaggerated manner^9^.

Together these hypotheses allow an examination of the biomechanical significance of the long internal projection of the incisor in diprotodonts during their most distinctive behaviour, gnawing.

## Methods

### Sample and model creation

One specimen of each of the following taxa was selected: *Hystrix cristata* (crested porcupine, Rodentia), *Rattus norvegicus* (brown rat, Rodentia), *Sciurus carolinensis* (grey squirrel, Rodentia), *Castor canadensis* (North American beaver, Rodentia), and *Daubentonia madagascariensis* (aye-aye, Primates). These were chosen based on the length of their incisor roots, the availability of information about their masticatory musculature, and the quality of the available microCT scans, as well as to ensure that the three extant suborders and the major muscle morphotypes (sciuromorph, myomorph and hystricomorph^26,27^) of rodents were represented alongside a non-rodent diprotodont.

Specimens were obtained and microCT scanned as part of previous projects^7,9,28,29^. No live specimens were used in this study. Further details on specimen scanning parameters and the institutions from which they were borrowed are given in Supplementary Table S1. Mandibles for all specimens were reconstructed virtually from microCT data using Avizo 8 (Thermo Fisher Scientific, Waltham, MA, USA). Isometric voxel dimensions ranged between 0.05 and 0.08 mm. All models had their unfused hemi-mandibles reconstructed, reflected and merged into a single mandible through reference to physical specimens, and by ensuring that, when merged, the mandibular condyles aligned with the glenoid fossae on the associated cranium. Incisors and bony material were segmented as separate materials and all holes and gaps that would usually represent cancellous bone or pulp were filled and treated as solid cortical bone and incisor respectively. Though representing cancellous bone as cortical bone significantly lowers strain (and therefore stress) magnitudes, it has been demonstrated that solid models produce patterns of strain and deformation almost identical to models with cancellous bone represented as a separate material^30–32^. All models were produced in this way, keeping any decreases in strain consistent between specimens. Furthermore, creating solid models enabled the variations in incisor length and in-filling of the alveolar space to be achieved in a repeatable manner between specimens.

**Figure 1:**
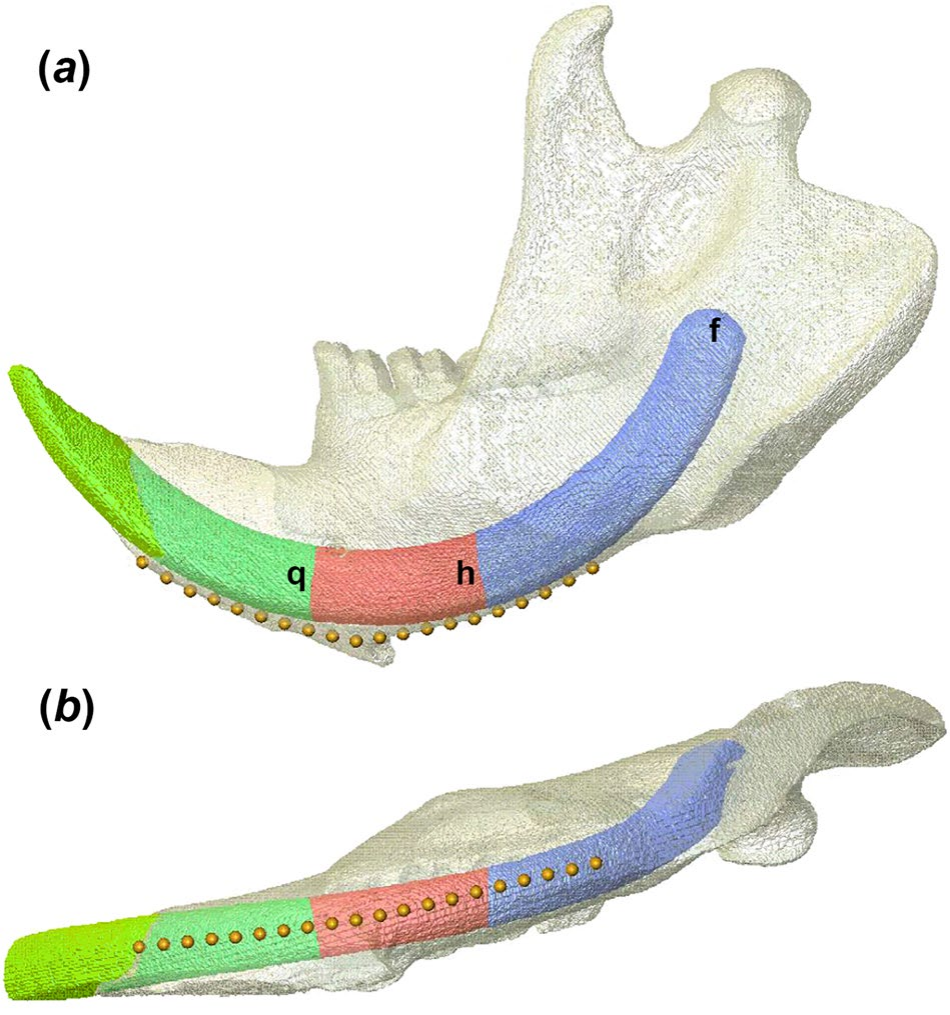
Model of *Castor canadensis* mandible in (**a**) lateral and (**b**) ventral view with landmarks placed for stress extraction. Lower case letters represent posterior extent of (f) full length, (h) half length, and (q) quarter length incisor.

Five models were made for each specimen, with variations in the length of the incisor root, and in the presence or absence of an incisal crypt (Fig. 1). The first model was made to attempt to represent the biological reality of the specimen with a full length incisor, while the second and third reduced the incisor root (defined here as all internal material posterior to the inferior alveolar margin) to 50% and 25% of its original length respectively. In these two altered models the removed incisor material was replaced with cortical bone. In models four and five, incisor root length was also shortened to 50% and 25%, but the deleted volume of incisor was left as an empty, air-filled crypt. The shortened lengths of the roots were determined by measuring anteroposteriorly along the outer, labial margin of the incisor, from the alveolar margin to the deepest distal portion of the root.

### Production of FE models

After segmentation of the mandibles was completed, the models were exported as bitmap stacks and converted into a mesh of eight-noded linear cubic elements by direct voxel conversion. This FE mesh was then imported into the non-commercial voxel-based FEA software VOX-FE^33^. All models were assigned the same material properties based on previously published data for rodent bone and teeth. Cortical bone was assigned a Young’s modulus of 17 GPa^28^ and Poisson’s ratio of 0.3^34^. The incisor was assigned a Young’s modulus of 55 GPA and a Poisson’s ratio of 0.32. These values were averages of the published values for rodent enamel and dentine^28^ as the two tissues were not distinguished during the segmentation process as a consequence of insufficient CT resolution in some specimens. Furthermore, it has been noted that representation of separate dental tissues in FE models has very limited impact on overall stress distributions^35^. Post-incisor dentition was not segmented as a separate material from bone as the focus of this study was directly on variation in mechanical strength of the mandible as a result of changes in incisor length.

Each model was loaded with forces representing the major muscles of mastication. The layers of the masseter are here referred to as superficial, deep, and zygomatico-mandibularis (ZM)^36,37^. The muscles applied to all specimens were the superficial masseter, deep masseter, ZM, temporalis, and medial pterygoid. In the crested porcupine two additional muscles, the lateral pterygoid and the infraorbital part of the ZM were added; and in the beaver the posterior masseter was added, whilst the deep masseter was separated into anterior and posterior portions^38^. The lateral pterygoid was included only in the crested porcupine as in other specimens the muscle properties were either not noted, or produced only very small forces (Supplementary Table S2). Muscle masses for the crested porcupine are known^36^, but as the fibre lengths were unknown, those of the beaver^38^, the closest available specimen in size, were substituted. The beaver is a sciuromorph, however, so fibre lengths for the ZM muscle were also used for the infraorbital portion of that muscle^36^. Where not already published, the physiological cross-sectional area (PCSA) of each muscle was calculated by converting muscle mass to volume (assuming a muscle density of 1.0564 g cm^−3^^39^) and dividing by fibre length. Muscle force estimates (Supplementary Table S2) were then calculated by multiplying PCSA by an intrinsic muscle stress value of 0.3 N mm^−2^^40,41^.

Muscle attachment sites were determined based on published descriptions of attachment sites and muscle origins^36,38,42–44^. Using Avizo, landmarks were placed at the centroid of each muscle attachment site on a virtual reconstruction of the cranium, and these landmarks were imported into VOX-FE to orient the muscle vectors. The models were aligned to the molar occlusal plane and constrained in all three axes at the left and right temporomandibular joints (TMJ). The tips of both incisors were constrained in the direction of the bite (Supplementary Fig. S1). This assumed that the direction of the bite was perpendicular to the occlusal plane of the post-incisor dentition, a common assumption in the analysis of rodent incisal biting with FEA (e.g.^28,29,45,46^).

### Model solution and analysis of results

FE models were solved using VOX-FE. Results were compared via a visual assessment of coloured contour plots representing von Mises stress distributions across the whole mandible. Quantitative comparisons of stress across the mandible were performed via the recording of von Mises stress values at each of 20 landmarks located along the ventral margin of the mandible (Fig. 1), the region of the mandible predicted to experience the greatest increase in stress in our first hypothesis. These were placed at equal intervals between the alveolar margin and the posteriormost point of the molar row. Strain distributions were analysed to determine whether hypothesis 2 was supported. With the mandible aligned to the occlusal plane of the post-incisor dentition, a perpendicular dorso-ventral slice was taken through the solved FE model immediately posterior to the molar row, for examination of the distribution of principal strains 1 and 3 in cross-section.

## Results

### Reduction of incisor length and patterns of stress

**Figure 2:**
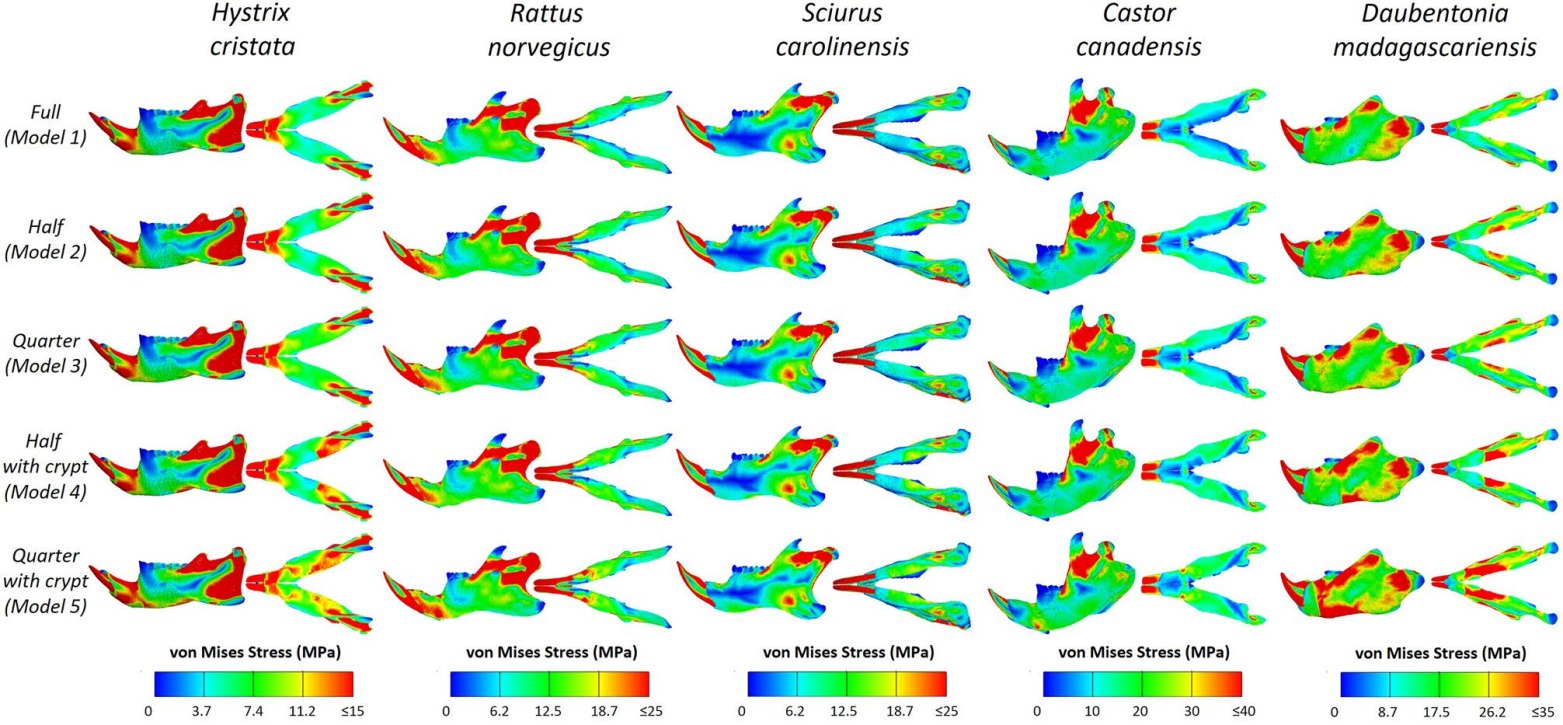
Contour plots of von Mises stress for all models and specimens in lateral and ventral views. Warmer colours represent areas of higher stress.

Figure 2 shows the distributions of von Mises stress across the mandibles of the five specimens in each of their five variant models. In general, stress distributions are very similar between variant models of the same taxon, with differences being mostly restricted to the ventral margin of the mandible, local to the incisor alveolus. Contour plots show a visible increase in von Mises stress along the ventral surface of the mandible as incisor length is reduced For all specimens there are clear increases in von Mises stress in areas closest to where the stiffer incisor material has been removed relative to the unmodified model.

**Figure 3:**
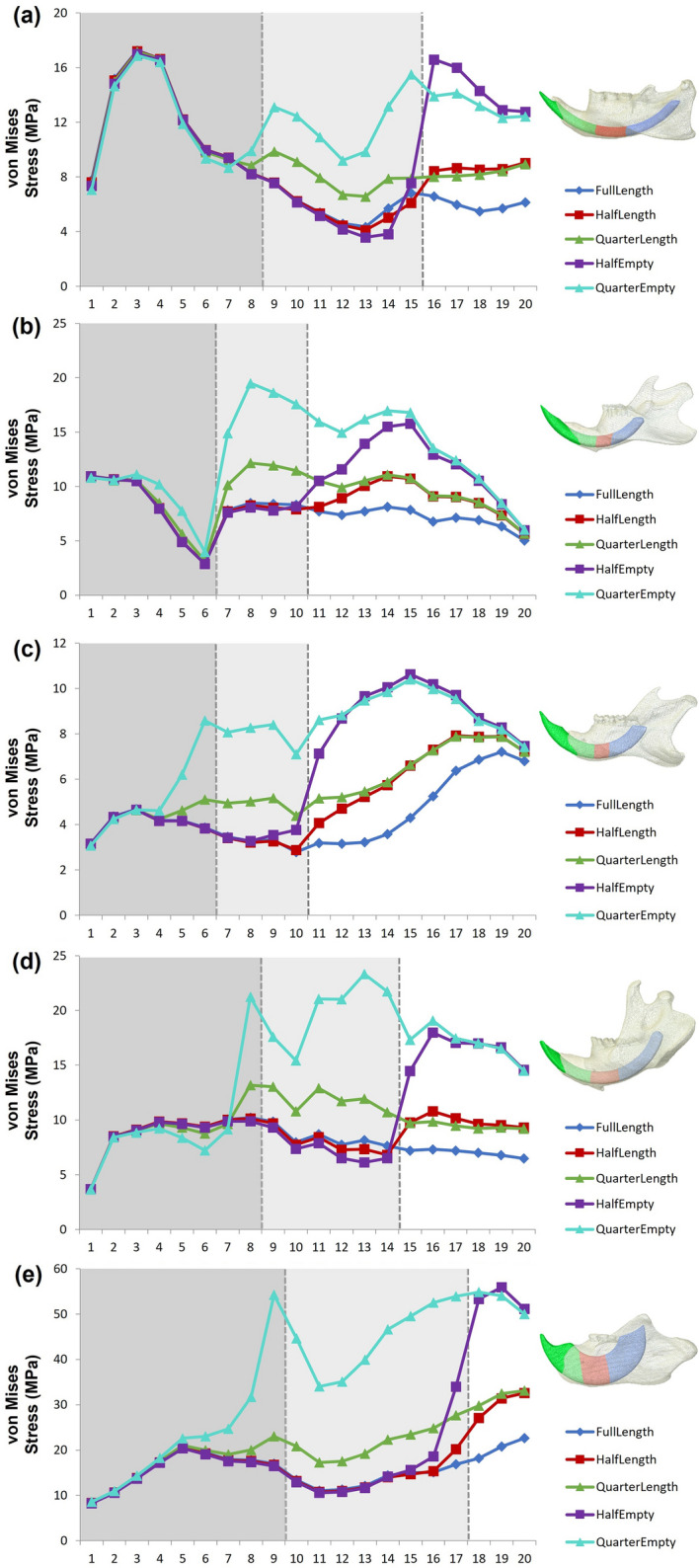
Values of von Mises stress at 20 points along the ventral mandibular margin in all five variant models for (**a**) *Hystrix cristata*, (**b**) *Rattus norvegicus*, (**c**) *Sciurus carolinensis*, (**d**) *Castor canadensis*, and (**e**) *Daubentonia madagascariensis*. Vertical dashed lines represent thresholds at which incisor material was removed in the variant models.

Plotting of the stress values extracted from the landmarks across the ventral surface of the mandible (Fig. 3) provides further detail: within all species, a dramatic increase in the magnitude of stress occurs at the threshold between the incisor and the region where incisor material has been removed or replaced. This is most pronounced within those that have had incisor material replaced with air, and is most evident in model 5 for all species. Typically, a steady increase in stress values is seen moving posteriorly along the ventral mandible. This increase levels out and occasionally reverses when the landmarks overlie areas of slightly thicker bone (as in the porcupine and brown rat, at around landmarks 4–7 and 3–6 respectively) before there is a dramatic change in stress as the landmarks overlie areas where material has been removed or replaced. Occasionally fluctuations occur from model to model within a species: for example in the porcupine model 4, stresses extracted from landmarks 14–16 suddenly transition from some of the lowest generated in any of the models of that specimen, to some of the highest. This coincides with the area at which the landmarks mark out the transition from solid incisor to the hollow crypt where stiff incisor material has been removed. Similarly, areas of thicker bone may cause stress to be consistently reduced in a particular region within a specimen, such as is visible in the area around landmark 10 for the beaver. At this landmark a sudden dip in stress occurs at the point where it overlies an area with a greater amount of bony material around the posterior end of the mandibular symphysis.

For all biting simulations, high stresses were exhibited around areas of muscle attachment, particularly on the thinner material of the coronoid process, and around the angular process. Visual examination reveals that even with the increased stresses as a result of the removal of incisor material, stresses along the ventral margin are still much lower than those seen at the muscle insertions.

Overall, it can be seen that hypothesis 1, that reduction in the length of the incisor root will result in an increase in stress across the mandible, was partially supported. However, increases in stress are mostly localised to areas close to the incisor alveolus.

### Strains in the region of incisor growth

**Figure 4:**
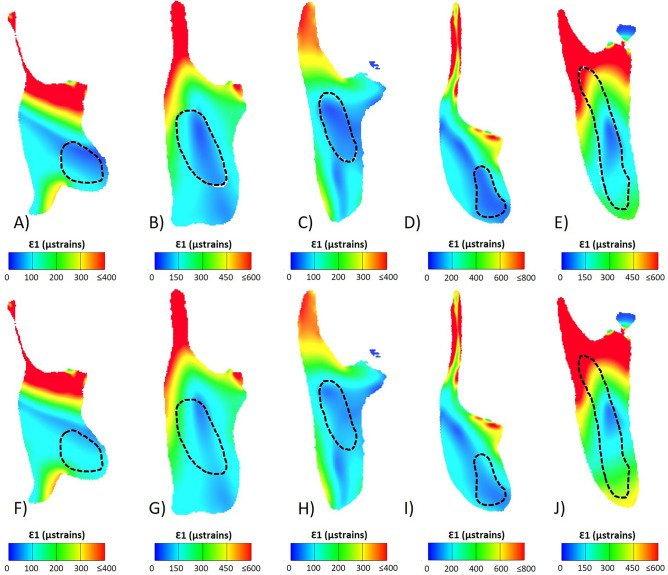
Principal strain 1 contour plots of a mandibular cross-section taken at the posterior most point of the molar row. The top row (**A**–**E**) shows model 1 (original, unmodified model), and the bottom row (**F**–**J**) shows model 3 (quarter length incisor, crypt filled with cortical bone). Species are (**A**,**F**) *Hystrix cristata*, (**B**,**G**) *Rattus norvegicus*, (**C**,**H**) *Sciurus carolinensis*, (**D**,**I**) *Castor canadensis*, and (**E**,**J**) *Daubentonia madagascariensis*. The dashed line indicates the position of the incisor. Warmer colours represent areas of higher strain.

**Figure 5:**
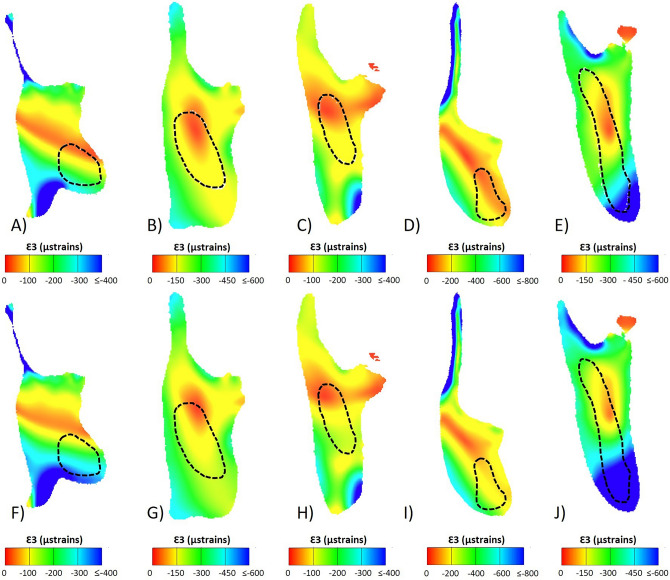
Principal strain 3 contour plots of a mandibular cross-section taken at the posterior most point of the molar row. The top row (**A**–**E**) shows model 1 (original, unmodified model), and the bottom row (**F**–**J**) shows model 3 (quarter length incisor, crypt filled with cortical bone). Species are (**A**,**F**) *Hystrix cristata*, (**B**,**G**) *Rattus norvegicus*, (**C**,**H**) *Sciurus carolinensis*, (**D**,**I**) *Castor canadensis*, and (**E**,**J**) *Daubentonia madagascariensis*. The dashed line indicates the position of the incisor. Cooler colours represent areas of higher strain.

With the mandible aligned to the occlusal plane of the post-incisor dentition and a perpendicular dorso-ventral slice taken through the solved FE model, the model was viewed in cross-section (Figs. 4, 5). From this view, models with reduced incisor lengths and bone-filled crypts (models 2 and 3) experienced somewhat low strains internally, with regions of higher compressive strains visible along the inferior portion of the cross-section, and higher tensile strains in the superior portion of the cross-section, often in regions associated with muscle insertion, such as the coronoid process. When comparing contour plots of the cross-sectional principal strains Ɛ1 and Ɛ3, the location of what appears to be the neutral axis of bending is apparent in all specimens. This region of lower strain covers an area overlapping with, or generally near to, the area occupied by incisor in model 1, but never fully overlaps or appears to correlate directly with it. There is also no clear region of lower strain that matches or approximates the shape of the incisor. Moreover, comparisons between variant models seem to indicate that replacing the incisor with bony material has a very limited effect on cross-sectional strain patterns (Figs. 4, 5). Thus, hypothesis 2, that the incisor grows through an area of naturally low strain, is not clearly supported.

### Patterns of stress in the aye-aye versus rodents

Plotting of the von Mises stress values extracted from the landmarks across the ventral surface of the mandible shows similar patterns across all five specimens (Fig. 3). However, in general the aye-aye experiences higher stresses in this region of the unaltered mandible (10–20 MPa) than do the rodents, with only the porcupine showing von Mises stresses over 12 MPa. The differences between the aye-aye and the rodents are then amplified in the variant FE models. Although, again, the patterns of stress along the ventral mandibular surface are largely similar across taxa, the relative increase in stresses in the aye-aye are much greater than in the rodents. This is particularly notable in model 5 (removal of three quarters of the incisor) in which a tripling of von Mises stress is seen at landmark 9 (the threshold between incisor material and empty alveolus), leading to stress values exceeding 50 MPa, over double that seen in any variant model of any of the rodent taxa. The lowest changes in von Mises stress are experienced by the brown rat, with a maximum increase in stress of just 130% relative to the unaltered model.

Overall, the aye-aye appears comparable to the rodent sample; although it experiences much higher stresses than the rodents as its internal incisor length is reduced, it produces patterns of strain similar to those observed in other examined diprotodonts. Thus hypothesis 3, that the non-rodent diprotodont aye-aye will perform in a mechanically similar, albeit more extreme, way to the rodents is supported.

## Discussion

### Reduction of incisor length and patterns of stress

Hypothesis 1 proposed that, by reducing the internal length of the incisor, an increase in stress across the mandible would be observed. This hypothesis was partially supported by the above results in that a reduction in the amount of internal incisor material resulted in an increase in stress local to the incisor alveolus in all five specimens. However, it was noted that von Mises stresses in regions further from the incisor (e.g. the coronoid, condyle and angular processes) were broadly constant across all variant models within each taxon. The increases in stress are most pronounced along the ventral margin of the mandible, an area which would be under high compressive strain in an arch-like structure. It has been proposed that the growth of the rodent mandible follows the course of the rodent incisor, producing an arch-like shape^3^. The mechanical significance of an arch is its ability to withstand a greater load than an otherwise materially equivalent horizontal beam can support. Vassallo^22^ found that as the mandible is exposed to three-point bending, its curved, arched shape reduces the bending moment by up to 25% more than in a beam-like mandible and argued this to be a functional adaptation for the resistance of forces encountered during gnawing. The curvature of the mandibular corpus, associated with the curvature of the underlying incisor, together with the increase in vertical height of the mandibular corpus are more effective ways of providing resistance to this sort of bending than increasing the medio-lateral thickness of the corpus^47,48^. It is possible these two features are not unrelated, as proposed by Druzinsky^3^. It may be, then, that the growth of a large internal incisor not only serves as a means of stress reduction along the ventral mandibular margin, by increasing the overall stiffness of the mandible, but also allows for the development of a morphology (an arch) that markedly reduces the moment of bending. The two in tandem may be key to the mechanical strength of the rodent-like mandible.

The long internal incisor length has been proposed to be relevant in the dissipation of forces along the length of the incisor and to the adjoining bone, thereby relieving stress in the mandible itself^18,19^. Teeth are composed of a composite of nearly inelastic materials and are among the most deformation resistant biological materials^49^. In the models tested here, the reduction in the length of the incisor reduces both incisor surface area through which to dissipate the forces encountered during incision and the volume of stiff material to resist bending. This predictably results in increased stress along the mandible in the areas previously occupied by the stiffer incisor material, notably across the ventral margin of mandible. That the amount by which stress increase was related to the volume of material in the model replaced with cortical bone or air (Fig. 3), suggests that the presence of greater quantities of more stiff incisor material plays a key role in maintaining the strength of the mandible as a whole during incision. Landry^50^ described how the embedded root of the upper incisor increases the contact between the tooth and skull, dissipating forces encountered during incision more effectively, thereby protecting the “tender growing end of the incisor” at its distal end. The apical portion of the incisor consists principally of more delicate, unmineralised tissues^4,18^. How these tissues are affected by the shortening of the incisor, and the role the impact upon them during incisal biting requires further investigation, including simulation of bites where the softer pulp material of these tissues is reflected in the FE models.

The presence of a greater volume of stiff incisor material may play a key role in the resistance of forces encountered during particularly demanding incisal biting, as rodents with longer incisors tend to exhibit diets that either regularly incorporate hard food items, or live in fossorial environments^9^. The beaver and the aye-aye exhibit some of the greatest increases in von Mises stress and also gnaw on resistant materials such as wood^5,51^. Compared to the other specimens in the study, and indeed many other rodents^9^, these two species exhibit far longer incisor roots relative to the amount of incisor protruding from the alveolus. Similarly, chisel-tooth digging rodents that require high bite forces to penetrate hard substrates have also been observed to possess incisors with notably deep roots^21,52^. The larger stress increases in these specimens may be reflective of a larger volume of tooth material that has been replaced by bone or air. However, the grey squirrel exhibits a maximum relative increase in stress similar to that seen in the beaver and aye-aye and does not have as relatively long an incisor as those two species, although it still reaches well past the molar row. Sciuromorphous rodents such as the grey squirrel have been shown in the past to exhibit very high incisor bite forces for their size^13,52^. The greater stress increases in the squirrel, beaver and aye-aye compared to the more generalist brown rat in the present study may suggest that a long internal incisor is particularly advantageous in the resistance of bone stress in species that habitually use their incisors to interact with mechanically resistant objects. Alternatively, the large stress increases may be a functional signal reflecting high incisor bite forces.

Despite the increases in stress related to removal of incisor material noted above, the stress changes from model to model are highly localised. Stress patterns in many parts of the mandible are seemingly unaffected by changes in incisor length, implying that, while it has some impact on stress, the length of the diprotodont incisor could be principally driven by factors other than bone stress reduction. Such drivers could include the rapid replacement rates seen in many rodents (full replacement of the lower incisors every 40–50 days in *Rattus norvegicus*^53^), which could require a long incisor to ensure that the tooth is fully mineralised by the time it erupts from the alveolus. Alternatively, there may be a developmental and geometric constraint that requires the incisor to have a long internal root in order that the erupted section emerges at the correct angle from the jaw^50^. Indirect evidence for this second theory is provided by chisel-tooth digging rodents which show longer, and more procumbent, incisors^54,55^.

### Strains in the region of incisor growth

Strain patterns in a mandibular cross-section were assessed qualitatively to determine whether the area that would have been filled by incisor is visible in the contour plots. Bending results in compressive stresses on one side of the bone and in tensile stresses on the other side, with a neutral axis in between the two that does not experience compression or tension during bending^56,57^. Visual assessment of the strain distributions (Figs. 4, 5) indicated that there was indeed an area of low strain in cross-section that overlaps at least partially with the area through which the incisor root would have grown. This overlap is not unsurprising as the incisor root in rodents is particularly large (particularly in the aye-aye), and as previously noted makes up a substantial percentage of the total volume of the mandible. Hypothesis 2 proposed that the area in which the incisor had been reduced and the crypt filled with cortical bone would experience lower strains than the area immediately around it, and that the location that would have been occupied by incisor material would be visually apparent. Though the path the incisor would have grown through in the mandible is not obvious from the strain contour maps, the maps demonstrate that the region does have somewhat lower strains. This could suggest that the incisor grows in an area of naturally lower strains; however the second hypothesis cannot be clearly supported, as this slightly less strained area never fully overlaps or correlates directly with the incisor root. Additionally, it is important to acknowledge that, even with the incisor material removed, the shape of the mandible remains the same^3,22^.

### Patterns of stress in the aye-aye versus rodents

Previous studies have demonstrated that the incisor morphology of the aye-aye is well-adapted to resist the forces encountered during gnawing behaviours, and that its specialised ecology has driven the evolution of an incisor morphology that is similar to, but more extreme than, that seen in rodents^9^. Thus, hypothesis 3 proposed that the non-rodent diprotodont aye-aye would perform in a mechanically similar way to rodents, but to a more extreme degree. The results of this study show that patterns of von Mises stress seen in the aye-aye closely resemble those of the rodents, exhibiting regions of high stress in similar regions of the mandible. However, the maximum relative increases in stress in the aye-aye were notably greater than in the rodent sample (250% compared to 200% in the squirrel and 185% in the beaver). The similarly large stress increases in the aye-aye and squirrel may be related to the convergence in mandibular shape seen in these two species^8^. Overall, it appears that the aye-aye has a rodent-like masticatory apparatus and form of diprotodonty that behaves in a mechanically similar way to that seen in rodents, especially squirrels, thus supporting hypothesis 3.

### Limitations and future work

As with all finite element studies, the models here are subject to a series of assumptions and simplifications to try and reflect a complex biological reality. In this study we did not model the PDL owing to limitations of scan resolution and a lack of consensus on the impact on strain distributions in FE models from the inclusion of PDL^28,58,59^. Furthermore, in reality, diprotodont incisors are only attached to the mandible on their labial side, whereas due to model resolution and the limitations of a voxel-based model the modelled incisor attaches to the mandible wherever voxels meet all along the internal length. However, both of these simplifications were consistent across all five models, allowing us to compare relative changes between variant models.

The fusion of the hemimandibles during the modelling process may have introduced some error into the FEA results. Rodents show varied symphyseal morphologies from very loose ligamentous connections (e.g. in rats^60^) to robust bony fusion (e.g. in beavers^38^). Soft tissue connections at the symphysis may provide some degree of shock absorbance^61^, thus reducing overall mandibular stress. Thus, the FE models produced in this analysis will have overestimated mandibular stiffness, and potentially stress^62^, in those taxa with reduced symphyseal fusion. However, given that overall stress patterns did not change greatly between variant models, and that our interpretations were based on relative changes between models of the same taxa, we believe that the representation of symphyseal morphology as complete bony fusion in all taxa is a simplification that will not have changed our overall conclusions.

Future work would benefit from the exploration of whether the crania of rodents and rodent-like diprotodonts produce similar results to more compliant mandibles. Janis et al.^20^ found that shortening the root of the upper canine of saber-toothed marsupial *Thylacosmilus atrox* (to match saber-tooth cat *Smilodon fatalis*) resulted in an increase in deformation, but that overall the effect on stress magnitudes was very minor. In that instance, other bony features of the skull such as the postorbital bar appeared to be more key to the resistance of forces when biting, rather than the deep tooth root, which appeared to be more important for anchoring the long canine^20,63^. It would be interesting to determine whether the enlarged upper incisors of rodents perform a similar role or if they assume greater importance in resisting masticatory forces.

### Conclusions

Here we examined whether the relatively stiff and elongated incisor, seen in rodents and aye-ayes, acts as a strengthening structure within the relatively less stiff bony mandible during incision. The FE models demonstrated that shortening the internal incisor root and replacing it with bony material or an empty space results in an increase in stress local to the incisor alveolus, but that the overall stress distribution across the mandible remained largely constant. This study found that the long incisor root may play a role in resisting bending forces, and may act in tandem with an arch-like mandibular shape to aid in strengthening of the mandible. However, the localised nature of stress reduction indicates that the principal driver of the elongate incisor may not be mechanical, and could be related to tooth wear or development. The incisor and mandible of the non-rodent diprotodont aye-aye appear to behave in a mechanically similar manner to those examined in rodents, particularly other hard-object biting species, albeit showing more extreme results and greater increases in stress as a consequence of modifications to its dental morphology. This result supports earlier findings that the aye-aye has converged upon a rodent-like mandibular and incisor morphology^7,9^ and finds that the aye-aye also resembles rodents in its biomechanical response to incisal biting.

## Supplementary Information

Supplementary Information.

## Data Availability

Surface reconstructions or original microCT scans of all specimens are available from http://www.morphosource.org (specimen numbers given in Supplementary Table S1).
