# Supplementary material for: The biomechanical significance of the elongated rodent incisor root in the mandible during incision
Source: Sci Rep. 2022 Mar 9;12:3819. doi: 10.1038/s41598-022-07779-z (PMC8907204; doi:10.1038/s41598-022-07779-z)
Supplement: Supplementary file 1 — Supplementary Information. [file 41598_2022_7779_MOESM1_ESM.pdf]

Species	Common name	Order	Family	Institution	Catalogue No.	Specimen	Voxel res mm	Date scanned	Where scanned	Morphosource DOI
Castor canadensis	American beaver	Rodentia	Castoridae	UMZC	E.1831	Osteological	0.0747	20/04/12	DEH	10.17602/M24089
Hystrix cristata	Crested porcupine	Rodentia	Hystricidae	UMZC	E.3406	Osteological	0.0791	20/04/12	DEH	10.17602/M24145
Rattus norvegicus	Brown rat	Rodentia	Muridae	DMBL	Rat4	Whole head	0.0539	21/09/09	DEH	10.17602/M23930
Sciurus carolinensis	Grey squirrel	Rodentia	Sciuridae	DMBL	Squirrel11	Whole head	0.0657	15/01/10	DEH	10.17602/M23931
Daubentonia madagascariensis	Aye-aye	Primates	Daubentoniidae	UMZC	E.8201.A	Osteological	0.0518	17/04/13	DEH	10.17602/M24097
**Abbreviations**										
DEH	Department of Engineering, University of Hull, UK									
DMBL	Department of Musculoskeletal Biology, University of Liverpool, UK									
UMZC	University Museum of Zoology Cambridge, UK									

Supplementary Table S1: Taxonomy, accession numbers and scanning details of specimens used in this study

Species	Data Source	Muscle	Fibre length (mm)	Muscle mass (g)	PCSA (cm3)	Force (N)
Hystrix cristata	Turnbull, 1970 (muscle masses) Cox & Baverstock, 2016 (fibre lengths)	Superficial masseter	28.1	11.9	4.01	120.26
		Deep masseter	27.6	4.9	1.68	50.5
		Infraorbital ZM	21.2	6.2	2.76	83.05
		ZM	21.2	4.3	1.92	57.6
		Temporalis	31.1	6.3	2.92	87.7
		Medial pterygoid	20	2.1	0.99	29.81
		Lateral pterygoid	11.7	2.2	1.78	53.39
Rattus norvegicus	Cox & Jeffery, 2011	Superficial masseter	25.3	0.53	19.83	5.95
		Deep masseter	15.4	0.89	58.35	17.5
		ZM	8.4	0.17	13.77	4.13
		Temporalis	20.8	0.69	31.85	9.56
		Medial pterygoid	8.2	0.21	24.81	7.44
Sciurus carolinensis	Cox & Jeffery, 2011	Superficial masseter	27.4	0.99	34.31	10.29
		Deep masseter	20.5	1.3	60.91	18.28
		ZM	10	0.31	29.66	8.9
		Temporalis	29.6	0.43	13.96	4.19
		Medial pterygoid	11.6	0.4	34.2	10.26
Castor canadensis	Cox & Baverstock, 2016	Superficial masseter	28.1	21.04	7.08	212.3
		Anterior deep masseter	39	7.79	1.89	56.8
		Posterior deep masseter	16.1	4.43	2.61	78.3
		ZM	21.2	10.19	4.56	136.8
		Posterior masseter	1.1	1.05	0.49	14.6
		Temporalis	31.1	19.35	5.89	176.6
		Medial pterygoid	20	5.61	2.6	79.7
Daubentonia madagascariensis	Perry et al, 2014	Superficial masseter	8.4	3.3	371.08	111.32
		Deep masseter	8.3	1.6	183.12	54.93
		ZM	9.3	1.9	194.3	58.29
		Temporalis	38.3	14.5	1060.45	318.14
		Medial pterygoid	5.9	1.6	256.63	76.99

Supplementary Table S2: Muscles of mastication applied to models, with details of fibre lengths, muscle volumes and masses, physiological cross sectional areas (PCSA) and force. Abbreviations: ZM, zygomatico-mandibularis.

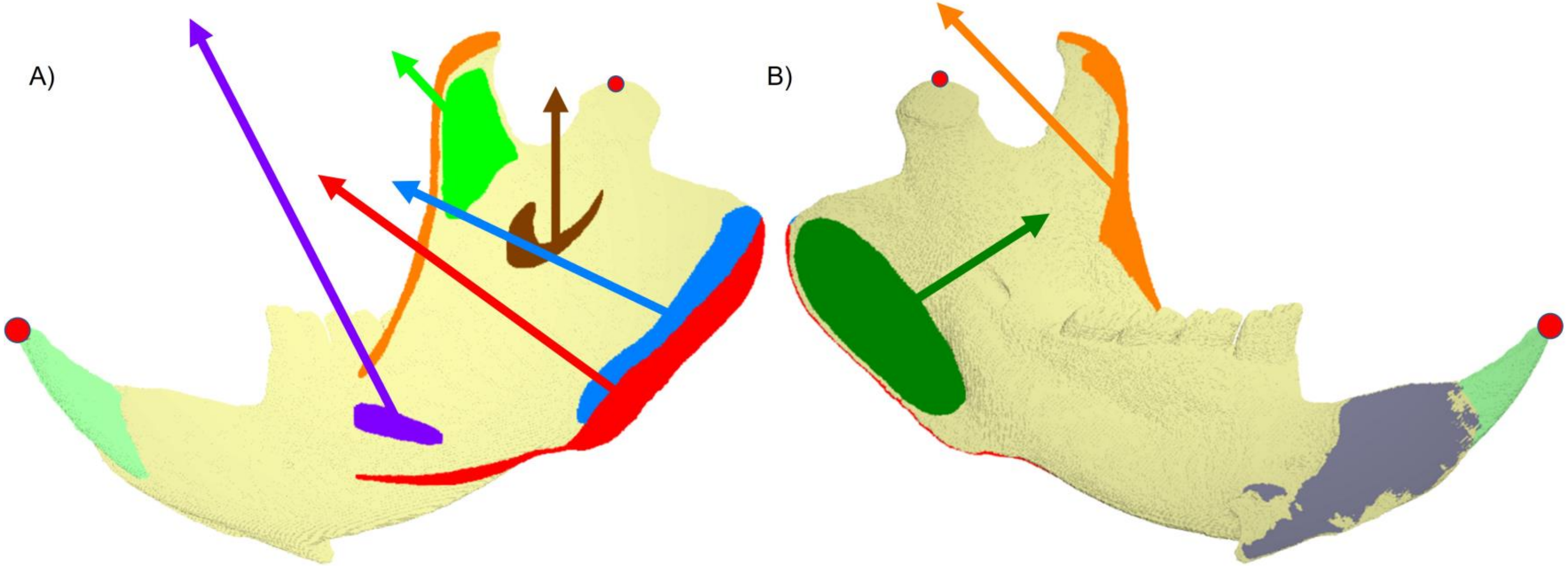

Figure S1: Free-body diagram of left-hemimandible of *Castor canadensis* in lateral (A) and medial (B) views, indicating muscle attachment sites (coloured zones) and their associated vectors (coloured arrows), and locations of constraints (red circles). Both the temporomandibular joints were constrained in all three axes, while tips of both incisors were constrained in the direction of the bite, perpendicular to the post-incisor dentition. Muscle attachments are the anterior deep masseter (purple), posterior deep masseter (blue), temporalis (orange), posterior masseter (brown), superficial masseter (red), zygomaticomandibularis (light green), and medial pterygoid (dark green).
